# The effect of breast cancer on personal income three years after diagnosis by cancer stage and education: a register-based cohort study among Danish females

**DOI:** 10.1186/s12889-015-1387-0

**Published:** 2015-01-31

**Authors:** Ingelise Andersen, Christophe Kolodziejczyk, Karsten Thielen, Eskil Heinesen, Finn Diderichsen

**Affiliations:** Section of Social Medicine, Institute of Public Health, University of Copenhagen, Øster Farimagsgade 5, P.O. Box 2099, 1014 Copenhagen K, Denmark; KORA, Danish Institute for Local and Regional Government Research, Købmagergade 22, DK-1150 Copenhagen K, Denmark; Rockwool Foundation Research Unit, Sølvgade 10, 2. tv, DK-1307 Copenhagen K, Denmark

**Keywords:** Breast cancer, Income, Longitudinal, Inequality

## Abstract

**Background:**

The purpose of this study was to investigate whether there is an association between stage of incident breast cancer (BC) and personal income three years after diagnosis. The analysis further considered whether the association differed among educational groups.

**Methods:**

The study was based on information from Danish nationwide registers. A total of 7,372 women aged 30–60 years diagnosed with BC, 48% with metastasis, were compared to 213,276 controls. Generalised linear models were used to estimate the effect of a cancer diagnosis on personal gross income three years after diagnosis, stratified by education and stage of cancer. The models were adjusted for income two years prior to cancer diagnosis and demographic, geographic and co-morbidity covariates.

**Results:**

Adjusting for income two years prior to cancer diagnosis and other baseline covariates (see above), cancer had a minor effect on personal income three years after diagnosis. The effect of metastatic BC was a statistically significant reduction in income three years after diagnosis of −3.4% (95% CI −4.8;-2.0), −2.8% (95% CI −4.3;-1.3) and −4.1 (95% CI −5.9;-2.3) among further, vocational and low educated women, respectively. The corresponding estimates for the effect of localised BC were −2.5% (95% CI −3.8; −1.2), −1.6% (95% CI −3.0; −0.2) and −1.7% (95% CI −3.7; 0.3); the latter estimate (for the low-educated) was not statistically different from zero. We found no statistically significant educational gradient in the effect of cancer stage on income.

**Conclusions:**

In a Danish context, the very small negative effect of BC on personal income may be explained by different types of compensation in low- and high-income groups. The public income transfers are equal for all income groups and cover a relatively high compensation among low-income groups. However, high-income groups additionally receive pay-outs from private pension and insurance schemes, which typically provide higher coverage for high-income workers.

## Background

It is well known that serious illness may have a detrimental effect on personal and household incomes. Seriously-ill people often have to leave the labour market, reduce working hours or change their job assignment due to the long-term effects of the disease itself or due to the side-effects the treatment has on physical and mental functioning and well-being [1,2]. The extent to which serious illness affects income depends on the welfare system and employment possibilities. Particularly in countries without universal health coverage and disability insurance, where social security is dependent on employment, people who are unemployed, sick-listed or without health insurance will have a high risk of experiencing severe economic consequences. This is in contrast to universal welfare societies [3], where welfare coverage is intended to provide economic security for all during illness. However, welfare societies also aim at improving the employment prospects of chronically-ill people, and participation in economic and social life will help them to maintain their living standards [4]. However, the employment rate of people with chronic illness differs in welfare states. Comparative studies have shown that people with chronic illness and low education have a lower employment rate in the UK and Denmark compared to Sweden and Norway [5,6]. For Denmark, this could be due to the Danish flexicurity system with a liberal hiring-and-firing procedure, which combined with relatively generous social security and active labour market policies makes it easy to fire chronically-ill persons. However, social benefits do not ensure that chronically-ill individuals are able to continue the same high standard of living. Whether there is a differential effect on income depending on the severity of the disease has not been well investigated.

Survival in breast cancer (BC) has improved steadily over the last 20 years. The five-year age-standardised survival rate has increased from 71% (95% CI 70; 72) in 1989–1993 to 83% (83; 84) in 2009–11. The annual incidence of BC among women aged 30 to 59 increased from 146.5/100.000 in 2000 to 149.6/100.000 in 2010, whereas the mortality decreased from 26.1/100,000 in 2000 to 18.2/100,000 in 2009. Consequently, the number of BC survivors has increased dramatically during the years 2000–2010 from 36,933 to 54,605 women (all ages) [7].

Previous Danish studies have revealed a 9% better overall survival rate from BC among women with higher education compared to women with lower education, although more affluent women have a higher incidence of BC. The social inequality in the burden of the disease, however, is not only generated by inequality in incidence, but also by inequality in the consequences of disease such as survival, disability and labour market participation [8]. Some studies have shown that the seriousness of the disease and type of treatment point towards lower employment rate after treatment [2]. A Danish study showed an education- and employment-adjusted impact of household income the year before cancer diagnosis, as well as an impact of co-morbidity on the risk of unemployment and disability pension [9]. The social consequences of BC have not been very well investigated, and the relatively few studies are impeded by weak research designs [10]. A recent systematic review reported that old age, low education, and low income before cancer were negatively associated with employment after cancer [1]. However, the review did not evaluate income after cancer.

The relatively few studies on cancer survivors and income after cancer diagnosis have reported contrasting results. A study from the US found that, compared to working controls, BC survivors experienced significantly larger reductions in annual earnings over the 5-year study period – primarily due to reduced work effort, not to changes in pay rates [11]. A Norwegian study found that BC survivors experienced a 14% reduction in earnings compared to employed females without cancer. Among all cancer types, the income reduction was significantly higher among metastatic cancer compared to local cancer, and low education was a risk factor for decline in income [12]. Conversely, a Swedish study did not find any effect on income (assessed as personal disposable income) three and five years post diagnosis; but that study did not investigate income in different educational groups [13]. A previous analysis of the data used in the present study did not find any significant effect of a BC diagnosis on income three years after diagnosis– either in general or among specific educational groups [14]. However, that study did not account for the severity of the disease, and it is possible that metastatic BC, for example, may have significant consequences for income. When stage at diagnosis was considered the results showed a significant negative effect on employment [15].

By means of the Danish registers, we investigated changes in individual income levels (measured as personal gross income) among females, aged 30–60 years, who survived at least three years after a BC diagnosis. We used cancer-free women as controls. We evaluated whether income three years after diagnosis, controlling for income two years before diagnosis, was associated with stage of cancer at the time of diagnosis. We also evaluated whether there was an interaction between stage and education that indicated an educational gradient in the consequences of cancer stage on income.

## Methods

### Study population

In this register-based cohort study, the cancer group (N = 7,372) included all women living in Denmark, who were diagnosed with incident BC in the period 2000–2006, who did not have any cancer before the incident BC, who were 30 to 60 years of age in the year of diagnosis, who survived at least to the end of the third year after diagnosis, and who did not receive disability pension or transitional benefits (indicating permanent withdrawal from the labour force) two years before the base year.

Cancer-free women were used as controls and were collected from the administrative registers covering all citizens in Denmark. For each of the base years 2000–2006, the control group consisted of females living in Denmark, 30 to 60 years of age, who survived at least three years after the base year and did not have a cancer diagnosis (either before or after the base year). For every year, we selected for analysis a random sample of 4% of the women meeting the criteria mentioned above, resulting in a total of 213,276 controls. The procedure for randomly selecting the control group ensured that no person was in the control group for two different base years.

We included women aged 30–60 years in the year of diagnosis for the following reasons: 1) BC before age 30 is very uncommon, 2) some people do not complete their education until after the age of 25, and 3) the age of retirement is 65 years. Since we were interested in the effect on income three years after BC, women should be at working-age at follow-up. We investigated the associations three years after BC diagnosis because during the first year or two, when women are undergoing intensive treatment, many (in particular white-collar workers), will receive their normal wages even during long-term absence for sickness because of collective agreements, while people with low income are well-supported by sick-leave benefits; therefore, we would not expect to find any large changes in income in the first three years. After three years, white-collar workers would typically have lost their previous job in cases of a significant loss of ability to work. It would be interesting to study more long-term effects on income, but this would require conditioning the analysis on long-term survival (e.g., five or ten years after diagnosis), resulting in a more selective sample and reduced sample size.

### Register data

The Danish civil registry number (CPR), a ten-digit code unique to every person living in Denmark, enables linkage between the CPR register with other register data on demographics, education, labour market status, income, municipality of residence, contact with general practitioners, hospitalisation, and purchase of prescription drugs.

### Data on cancer

Data on cancer was obtained from the Danish Cancer Registry, which contains data on the incidence of cancer throughout Denmark since 1943. The Cancer Registry contains information related to the personal characteristics of the patient diagnosed with cancer and tumour characteristics. For this investigation, we included BC patients (ICD 10 code C50). The BC patients were dichotomised into a group of 3,769 women with cancer at a localised stage (had not entered the lymphatic system) and a group with 3,603 women with regional lymphatic spreading or any other kind of metastasis. We combined regional spread with other kinds of metastasis, since only very few BC survivors had other kinds of metastatic BC at the time of diagnosis.

### Socioeconomic position (SEP)

SEP was defined by education. Information on education was obtained from registers for the baseline year. Education was classified into three categories according to the International Standard Classification of Education (ISCED) system (UNESCO 1997): (1) compulsory education, i.e., up to 10 years of education (ISCED level (0–2), (2) vocational education, i.e., up to 11–12 years of education (ISCED level 3), (3) further education, i.e., 13 or more years of education (ISCED level 4–6).

### Data on income

We used the personal gross income comprising the sum of the main income types: earnings, capital income, social benefits, and other transfers including private insurance payments released when people are diagnosed with a “critical” illness such as cancer. Data on income for each study participant was obtained from registers for each of the years from five years before baseline to three after baseline. Income was adjusted for inflation.

### Confounders

In the analyses, adjustments were made for control variables measured at least 2 years before the base year except for demographic and geographic variables, which were measured in the base year.

### Demographic variables

*Age* was included in the analyses as 30 one-year categorical variables for each age from 30 to 60 years of age. Information on *family type* was defined as single with children, married with or without children, cohabiting with or without children. *Children in the family* were categorised as no child or children 0–2, 3–6, 7–9 or 10–14 years of age. Information on *ethnicity* was dichotomised as ‘Danish-born yes/no’.

### Geographic variables

Since cancer prognosis could be affected by the *regional* health service system, we controlled for the five regions in Denmark as well as the *size of the municipality* in which the study participants lived, divided into Central Copenhagen, Copenhagen suburb, municipalities with >100, 40–99, 20–39 and <20,000 inhabitants. *Urbanisation* was divided into three groups: > 50%, 33-50% and <33%.

### Labour market status

The basic variables for labour market status were defined as the most important type of income during the year. The categories included wage earner, self-employed, unemployed and ‘out of the labour force’. The last category consisted mainly of people receiving social assistance, sickness benefits, disability pension, or early retirement benefits or people being supported by their spouse and a small share were students. We controlled for labour market status, income, earnings and labour market experience from two years before the base year and the change in these variables from five years before to two years before the base year.

### Health indicators

We included contacts with the healthcare system 2–5 years prior to baseline. From the hospital discharge register, we obtained information on *hospitalisation* for both inpatients and outpatients. Numbers of contacts with the GP were collected through the health insurance register; and, from the *prescription register*, we included purchases of the main relevant types of prescription drugs related to medical conditions such as hypertension, heart disease, mental disorders, Parkinson’s disease, osteoporosis, asthma, bronchitis, thyroid disorders, epilepsy, and headache.

### Endpoint: income three years after diagnosis

To investigate whether income was affected by the cancer diagnosis, we use as endpoint income three years after diagnosis (base year), and we control in the analysis for the variables discussed above, including income two and five years before diagnosis and interaction terms between lagged income and levels of education.

### Statistical methods

We modelled the conditional expectation of income (*y*) in time t + 3, i.e., 3 years after the base year/diagnosis, given a set of explanatory variables (*x*), including variables for breast cancer and control variables. A generalised linear model (glm) with log link and Poisson distribution was used to account for the highly-skewed distribution of Income (*y*). The conditional expectation was modelled as

$$ E\left(y\left|x\right.\right)= exp\left(x\beta \right) $$

in which *β* is a vector of parameters. The model assumes that the variance of *y* given *x* is equal to the conditional mean. Therefore, it was necessary to estimate a covariance matrix for the estimator, which is robust to the presence of unknown forms of heteroskedasticity. We used the White sandwich estimator. The advantage of this model was that we could easily compute effects in percentages on the dependent variable of changes in covariates. As we were interested in estimating the joint effect of education and cancer stage, we included interaction terms between education levels and cancer stage in the model. The relative change in the average value *y* related to a change of covariate *x*_*k*_ is equal to exp (*β*_*k*_)-1. For small values of *β*_*k*_, we have exp(*β*_*k*_) − 1 ≈ *β*_*k*_ and, in this case, changes in percentages were read directly from the coefficients of the model.

We estimated four models to investigate the association between cancer and gross income in year t + 3 (three years after diagnosis/base year). The first model was adjusted for age, year of diagnosis, children at ≥30 years of age, family structure, number of children, education, geographical variables, income in year t-2 (two years before the base year) and its interaction with education, and the change in income from year t-5 to year t-2. In this model, the stage of the disease was not taken into account, i.e., the *stage* vector was replaced by a single dummy variable to indicate BC diagnosis (yes or no). In the second model, we further adjusted for the stage of the disease as well as all the interactions between stage and education. The third model was further adjusted for co-morbidity measured by previous hospitalisation, consumption of prescription drugs and GP contacts, labour market status at time t-2, and changes in labour market status from t-5 to t-2. Lastly, the fourth model also adjusted for labour market attachment at time t + 3 (i.e., in the year of follow up). The goodness-of-fit of the models was measured by the RMSE.

### Ethics statement

The study was approved by the Danish Data Protection Agency. The register data were analyzed at Statistics Denmark. At Statistics Denmark the original CPR-numbers are replaced by other personal identification numbers in order to make the persons in the dataset anonymous to the researchers. Consequently, ethical approvals are not necessary.

## Results

Table 1 shows important baseline data for the study population grouped by cancer status: localised cancer, metastatic cancer, and control group. Results for the last two groups are age-standardised to the population with localised cancer. Without standardisation, women in the control group are on average about 6 years younger than women in the two cancer groups, which have very similar age distributions. Income in t-2 is about DKK 9000 (approx. $1500) higher among those, who get cancer two years later, and their level of education is also a little higher (37% have higher education compared to 33% for the control group), whereas the risk of being out of the labour force is very similar across groups. While income increases by about DKK 5000 on average from t-2 to t + 3 for the control group, it decreases by about DKK 1000 and 4000 for the two cancer groups. Thus, compared to the control group, the average income loss for the groups with localised and metastatic cancer is about DKK 6000 and 9000, corresponding to about 2.5% and 3.7% of pre-cancer income. After age-adjustment, there are no important differences between the three groups in terms of marital status at baseline. However, the proportion of women having no child at age 30 is 3–4 percentage points higher in the two cancer groups. Health 2–5 years before diagnosis seems to be worse for the cancer groups, especially the group with localised cancer, and especially with respect to use of drugs for mental health and yearly GP contacts. Differences in the proportion of women who used prescription drugs for somatic diseases and the proportion hospitalised for any diagnosis except obstetrics (in the period from t-5 to t-2) were smaller. The significantly younger mean age in the control group requires all analyses to be age-adjusted in order to make the three groups comparable.

**Table 1:** **Characteristics of the 7372 Danish breast cancer women (by cancer stage) and the 213,276 controls**

	**Cancer survivors**		**Cancer-free**
	**Localized**	**Metastatic**	**Control group**
N	3769	3603	213,276
Age, years, mean	50.47	50.47	50.47
Danish, percent	95	95	95
*Civil status*			
Married, percent	70	70	69
Single, percent	23	21	22
Cohabiting, percent	7	8	9
*Children*			
Proportion with no children at age 30, percent	27	28	24
*Education*			
Compulsory education, percent	26	27	29
Vocational education, percent	37	36	37
Further education, percent	37	37	33
*Income(gross income)*			
Change in income t-2 to t + 3, 1000 DKK, mean	−1.19	−3.87	5.36
Income t-2, 100,000 DKK, mean	2.41	2.39	2.31
Out of labour force t-2, percent	6	7	7
*Health indicators*			
Hospitalization 2–5 years before baseline, percent	47	46	45
Drugs for somatic diseases 2–5 years before baseline, percent	69	67	66
Drugs for mental health 2–5 years before baseline, percent	23	21	18
GP contacts 2–5 years before baseline, number, mean	6.42	6.21	6.04

Note: All figures for metastatic cancer survivors and the cancer-free control group are age standardized to the population with localized cancer.

Table 2 shows the estimated effects of key explanatory variables on individual gross income three years after incident BC. As shown in model 1 (with adjustment for demographics, education and lagged income), BC patients generally lost about 2.7% in income compared to the healthy population. The interaction terms between the cancer dummy (‘cancer yes/no’) and vocational and further education, respectively, are very small and not statistically significant. No statistically significant educational gradient was observed.

**Table 2:** **The effect of breast cancer and key control variables on individual gross income three years after the base year (t + 3)**

	**Model 1**		**Model 2**		**Model 3**		**Model 4**	
	**β**	**95%** **CI**	**β**	**95%** **CI**	**β**	**95%** **CI**	**β**	**95%** **CI**
Cancer vs healthy	−0.027	[−0.041,-0.012]						
Localized vs healthy			−0.015	[−0.036,0.006]	−0.017	[−0.038,0.003]	0.015	[−0.004,0.034]
Metastatic vs healthy			−0.040	[−0.060,-0.020]	−0.041	[−0.060,-0.023]	0.010	[−0.007,0.027]
Vocational vs Compulsory edu.	0.164	[0.151,0.178]	0.164	[0.151,0.178]	0.102	[0.089,0.114]	0.048	[0.036,0.060]
Further vs Compulsory edu.	0.368	[0.356,0.381]	0.368	[0.356,0.381]	0.282	[0.269,0.295]	0.202	[0.191,0.214]
Vocational education *Cancer	−0.001	[−0.020,0.017]						
Further education *Cancer	−0.002	[−0.020,0.016]						
Vocational edu. *Localized			−0.010	[−0.035,0.016]	0.001	[−0.024,0.026]	−0.013	[−0.036,0.010]
Vocational edu. *Metastatic			0.007	[−0.018,0.033]	0.013	[−0.011,0.038]	−0.006	[−0.029,0.016]
Further education *Localized			−0.007	[−0.033,0.018]	−0.008	[−0.032,0.016]	−0.030	[−0.052,-0.007]
Further education *Metastatic			0.005	[−0.020,0.029]	0.007	[−0.017,0.030]	−0.024	[−0.045,-0.002]
Income t-2	0.334	[0.329,0.340]	0.334	[0.329,0.340]	0.275	[0.270,0.280]	0.248	[0.243,0.253]
Vocational edu. *income t-2	−0.043	[−0.049,-0.037]	−0.043	[−0.049,-0.037]	−0.027	[−0.033,-0.022]	−0.010	[−0.015,-0.005]
Further education *income t-2	−0.092	[−0.097,-0.086]	−0.092	[−0.097,-0.086]	−0.072	[−0.077,-0.066]	−0.047	[−0.052,-0.042]
Out of labour force t + 3							−0.503	[−0.509,-0.497]
N	220648		220648		220648		220648	
RMSE	89.31		89.31		76.13		71.10	

Model 1: adjusted for age, children at age 30, family structure, number of children, year, income in year t-2 and t-5, education, education *lagged income, cancer and education *cancer.

Model 2: in addition adjusted for cancer stage and education*cancer stage.

Model 3: in addition adjusted for previous hospitalization, drugs, geography and labour market attachment time t-2 and t-5.

Model 4: in addition adjusted for labour market attachment time t + 3.

In model 2, the cancer dummy was replaced by the BC stages ‘localised’ and ‘metastatic’. For the group with only compulsory education, the consequence of metastatic cancer was an income loss of 4.0% (95% CI-0.060; −0.020); the income loss associated with localised cancer was less than half of this and insignificant: 0.015 (−0.036; 0.006). The point estimates of the interaction terms between BC stage and education indicated that the income loss of metastatic BC was a 0.5-0.7 percentage point smaller for those with a vocational or further education (compared to compulsory education) – that is, the income loss was 3.5% for women with a further education (−0.040 + 0.005) and 3.3% for those with vocational education. For localised cancer, the point estimates indicated an income loss of 2.5% for vocational education and 2.2% for further education. However, these differences between education groups were small, and none of interaction terms between cancer stage and education were statistically significant.

Model 3 additionally adjusted for baseline labour market attachment and health (in t-5 to t-2). The estimated income consequences of cancer were almost identical to those of model 2. As expected, the inclusion of controls for lagged labour market attachments implied smaller coefficients of the “main effects” of education and lagged income.

Model 4 was further adjusted for being out of the labour force at follow-up, i.e., in year t + 3. In this case, the effect of BC diagnosis was insignificant. The interaction terms between further education and localised and metastatic cancer were significant and negative. However, the point estimates for the cancer effects for this education group was only about −1.5 percent and insignificant for metastatic cancer and marginally significant for localised cancer. Thus, the small income loss due to cancer (of up to 4.1 percent) according to model 3 appears to be largely explained by an increased risk of leaving the labour force.

Table 3 summarises the estimated effects of BC on income by cancer stage and education based on the coefficients from model 3 in Table 2. Income losses as a consequence of BC were small, but statistically significant (with the exception of the localised cancer group with only compulsory education). Point estimates indicate that effects are a little larger for metastatic than for localised BC, but the differences were not significant. As discussed above, we observed no significant educational gradient in income effects either for localised or for metastatic cancer, and the point estimates did not indicate a clear pattern. The largest relative income effect was for women with metastatic BC with only compulsory education, for which the estimated income loss was 4.1 percent (95% CI −5.9; −2.3).

**Table 3:** **The percentage effect of stage of cancer on individual gross income (in year t + 3) by education, and 95**% **confidence intervals**

	**Compulsory education**	**Vocational education**	**Further education**
Localized vs healthy	−1.7	−1.6	−2.5
	[−3.7,0.3]	[−3.0,-0.2]	[−3.8,-1.2]
Metastatic vs healthy	−4.1	−2.8	−3.4
	[−5.9,-2.3]	[−4.3,-1.3]	[−4.8,-2.0]

Note: these changes are computed as the changes in percent in income for a given education level and are based on the coefficients of model 3 of Table 2. For example, compared to a woman without cancer and with vocational education the change of the average value of income for a woman with a localized cancer and the same education level is equal to exp(−0.017 + 0.001)-1 = −0.016. The 95% confidence intervals are computed using the delta method.

## Discussion

This large longitudinal register-based study revealed that, BC has only a minor impact on income three years after diagnosis. The largest effect was observed for metastatic cancer, but differences between effects of localised and metastatic cancer were not statistically significant. We did not observe a statistically significant educational gradient in the effect of BC on income, either for localised or metastatic cancer.

To our knowledge, this is the first study to investigate the association between stage at BC diagnosis and income. Previous studies of BC and income after diagnosis did not include measures of the severity of disease. Whereas a Norwegian study found overall negative income effects for all cancers, a Swedish and Danish study found no effect of BC on income [12-14]. The contrasting results may be due to differences in the specification of the analyses or, for the rather homogenous Nordic countries, differences in the welfare societies. The Norwegian results could be due to the inclusion of all types of cancer and cancer diagnosed over a long period of time, 1992–2000, with results potentially affected by improvements in treatment during this period [12]. The insignificant estimates for Sweden might be explained by the social insurance system, which guaranteed sickness compensation for a much longer period than in Denmark and Norway at the time of the study [13].

In this study, we expected that cancer stage would be important for the effect of BC on income and for the educational gradient in this effect. It is often hypothesised that the inverse social gradient in survival after BC is due to earlier diagnosis among more affluent women. However, the results in Table 1 do not point towards any educational differences between localised and metastatic BC among ≥3-year survivors.

The income loss due to cancer seems to be explained by a negative effect of cancer on employment. Table 2, models 3 and 4, show that, when controlling for labour market attachment at three years after diagnosis, the difference in income between women with metastatic BC and healthy women (for the low-educated group) changes from −4.1% to 1.0%. These results are consistent with another study from the same population showing significant effects of cancer stage on leaving the labour force three years after diagnosis [Thielen K, Kolodziejczyk C, Andersen I, Heinesen E, Diderichsen F: Cancer stage and socioeconomic differences in consequences of cancer on labour market participation. 2014 submitted].

It seems surprising that we only found a small effect of cancer stage on income, as other studies based on the same population reported significant negative effects on employment [Thielen K, Kolodziejczyk C, Andersen I, Heinesen E, Diderichsen F: Cancer stage and socioeconomic differences in consequences of cancer on labour market participation. 2014 submitted]. One explanation could be the Danish welfare system with relatively generous social benefits – in this case, primarily disability pension and extended sickness benefits, which provide high coverage, especially for low-income workers. The basic social benefits are 90% of minimum wage with a general upper threshold negotiated between organisations representing employers and employees. Another reason is that persons with substantial loss of working ability will receive insurance pay-outs from labour market pension schemes or private pension or insurance schemes. These schemes typically provide a percentage of previous earnings and will often provide higher coverage for high-income workers. This explains why highly-educated females maintain their income level after cancer.

Even though BC survivors only have to struggle with the economic consequences of the disease to a minor degree, a number of them still have reduced quality of life related to side-effects of cancer treatment, such as oedema, general fatigue, anxiety, and depression, which may result in some leaving the labour market several years after diagnosis [15,16].

### Strengths

The strengths of the study include the large sample size, longitudinal and population-based design, and the use of unique Danish registers containing yearly updated information on, e.g., income and labour market status. For this study, we used individual gross income. It may be discussed whether income before or after tax is most relevant. We ran the same analyses using personal income after tax instead of gross income. Since the results were similar and gross income is more comparable across educational groups, we present the results for gross income. We controlled for income and labour market status 2–5 years before diagnosis and, additionally, for co-morbidities based on hospitalisation, drug use and GP contacts 2–5 years before diagnosis, thus reducing the risk of residual confounding. As opposed to a number of studies that categorised all types of cancer together, we focused on BC and, therefore, obtained a clear picture of the impact of BC diagnosis on income in BC survivors.

### Limitations

One of the limitations of our study was that the control group was not comparable to the cases with respect to age. However, since the control group was very large and we were able to control very precisely for age differences, this is not an important limitation for our results. Another limitation is that we did not have data for whether jobs before the base year were physically demanding. Such jobs may be difficult to return to after BC. There might, therefore, be different effects of BC on income for women with different types of jobs (which are not fully controlled for by education). Another limitation is generalizability to populations outside Denmark. Women in Denmark are better supported during sick leave or entitled to disability pension compared to cancer patients in most other countries. However, even in this universal welfare society, it is a surprise that BC has such a small impact on income after three years. One explanation could be that BC has a relatively good prognosis, and the majority of women are able to continue working even during treatment, which could explain the minimal financial impact of BC. Consequently, the results may not be generalizable to other types of cancer.

## Conclusion

This register-based study of Danish BC survivors aged 30–60 years revealed that stage of cancer had only a minor effect on income three years after diagnosis after controlling for income two years before diagnosis. We did not find any significant social gradient in the effects of cancer on income. The very small negative effect of BC on income in Denmark may be explained by a relatively high compensation of welfare payments among low-income groups and pay-outs from private pension or insurance schemes, which typically provide higher coverage for high-income workers.

## Abbreviations

BC: Breast cancer
CI: Confidence intervals
DKK: Danish Kroner
GP: General practitioner
ICD: International classification of diseases
ISCED: International Standard Classification of Education
